# A Recombinant OMV-Based Vaccine Elicits Potent Protective Immunity Against *Pseudomonas aeruginosa*

**DOI:** 10.3390/vaccines14060518

**Published:** 2026-06-09

**Authors:** Jiannan Li, Guangyu Qi, Mingyue Cao, Zixian Wang, Gejin Lu, Xulong Lang, Feng Wei, Tiancheng Lu, Lingwei Zhu, Xiuran Wang

**Affiliations:** 1 College of Life Sciences, Jilin Agricultural University, Changchun 130118, China; lijiannan0102@163.com (J.L.); 19904387173@163.com (G.Q.); 13591320590@163.com (M.C.); 13756170500@126.com (F.W.); tiancheng@jlau.edu.cn (T.L.); 2 Key Laboratory of Jilin Province for Zoonoses Prevention and Control, State Key Laboratory of Pathogen and Biosecurity, Academy of Military Medical Sciences, Changchun 130122, China; ytszxc@163.com (Z.W.); lgj_jlu@126.com (G.L.); langxulong@foxmail.com (X.L.); 3 School of Laboratory Animal & Shandong Laboratory Animal Center, Shandong First Medical University & Shandong Academy of Medical Sciences, Jinan 250117, China

**Keywords:** outer membrane vesicles, antigen delivery, immunogenicity, vaccine

## Abstract

Background: This study aimed to construct a recombinant *Pseudomonas aeruginosa* outer membrane vesicle (OMV) vector vaccine delivering *pcrV* and compare the immunological impacts of OMVs as carriers versus as adjuvants. Methods: The recombinant plasmid pBBRMCS5-*pcrV* was constructed and transformed into *P. aeruginosa*. Recombinant OMVs (OMV_PcrV_) were prepared via ultracentrifugation and characterized in terms of their morphology and particle size by means of transmission electron microscopy (TEM) and nanoparticle tracking analysis (NTA). After a biosafety evaluation, mice were intramuscularly immunized with PcrV or OMV_PcrV_, followed by a booster immunization on day 21. On day 42, the mice were challenged subcutaneously and intranasally with PAO1. Bacterial loads in tissues and blood, pulmonary T-cell subsets, and serum antibody levels were assessed. Results: The recombinant plasmid was successfully constructed, and Western blotting confirmed the delivery of PcrV into OMVs. TEM revealed typical spherical nanostructures, and NTA showed a median particle size of 127.4 ± 5.3 nm. Upon subcutaneous challenge, the OMV, OMV_PcrV_, and OMV + PcrV groups all achieved 100% protection. Both the OMV_PcrV_ and OMV + PcrV groups exhibited increased CD4^+^ and CD8^+^ T-cell counts and higher induction levels of specific IgM, IgG1, and IgG2a antibodies. The OMV_PcrV_ group showed superior clearance of respiratory bacterial colonization and reduced inflammatory injury compared with the PBS control group. Conclusions: The constructed vector successfully delivered the PcrV antigen, and the OMVPcrV vaccine induced effective immune responses. Compared with wild-type outer membrane vesicles (OMVs) and the strategy of directly mixing free PcrV antigen with OMVs (OMV + PcrV), the recombinant OMV_PcrV_ vaccine exhibited superior immunoprotective efficacy in terms of bacterial clearance and tissue protection, providing experimental evidence for the development of a *Pseudomonas aeruginosa* vaccine.

## 1. Introduction

*Pseudomonas aeruginosa* is a Gram-negative bacillus belonging to the genus *Pseudomonas*. It is widely distributed in nature and can be found in soil, sewage, and air, as well as on or in the normal human skin, respiratory tract, and intestinal mucosa. It is a common opportunistic pathogen [1,2]. *P. aeruginosa* is a major cause of pulmonary infections in patients with cystic fibrosis, non-cystic fibrosis bronchiectasis, and chronic obstructive pulmonary disease, and it is a frequent pathogen responsible for infections in burn victims, organ transplant recipients, and intensive care unit patients [3,4]. This bacterium exhibits both intrinsic and acquired resistance to multiple antibiotics, posing significant challenges for treatment [5]. Therefore, vaccine development may represent a strategy to control *P. aeruginosa* infection and reduce antibiotic resistance. Since the 1960s, at least 60 vaccines against *P. aeruginosa* have been under investigation. Based on their different technological platforms, they can be categorized into component vaccines, subunit vaccines, live attenuated vaccines, whole-cell inactivated vaccines, and vector vaccines [6]. Among these, three candidates have entered clinical trials, but all have failed, and no vaccine against *P. aeruginosa* has been approved for marketing to date [7].

In recent years, outer membrane vesicles (OMVs) have garnered significant attention as a vaccine platform against bacterial pathogens. OMV-based vaccines against *Neisseria meningitidis* serogroup B [8] and *Haemophilus influenzae* type b [9] have been successfully developed and licensed. OMVs are nanosized spherical structures (20–300 nm) with a lipid bilayer that are spontaneously secreted by Gram-negative bacteria during growth [10]. These vesicles enclose periplasmic components and are formed through a blebbing process during the active growth phase, rather than as a result of bacterial lysis [11]. OMVs carry multiple pathogen-associated molecular patterns (PAMPs), such as endotoxin (lipopolysaccharide, LPS), peptidoglycan (PG), and bacterial DNA, which can bind to host pattern recognition receptors (PRRs) to initiate innate immune and inflammatory responses [12]. As non-replicative nanoscale particles, OMVs facilitate the uptake and presentation of antigens by antigen-presenting cells (APCs) without causing the associated diseases. Studies have indicated that OMVs remain stable under high-temperature and various chemical treatments [13]. The unique structure and functions of OMVs endow them with natural adjuvant properties [14], positioning them as a versatile platform for vaccine development. In recent years, the convergence of synthetic biology and nanobiotechnology has given rise to the emerging field of engineered outer membrane vesicles. By allowing antigens to be fused with different carrier proteins or peptides and expressed in the bacterial outer membrane or periplasm, these approaches enable targeted localization of antigens to the OMV outer membrane or lumen [15], thereby achieving targeted delivery. Several studies have described the expression of heterologous antigens by means of fusion with certain membrane proteins, such as the autotransporter hemoglobin protease (Hbp) [16,17], cytolysin A (ClyA) [18,19], and outer membrane protein A (OmpA) [20]. In addition, antigens can be bioengineered to target the lumen of OMVs, as exemplified by the fluorescent protein GFP [21] and *Streptococcus pneumoniae* protein PspA [22]. The type III secretion system (T3SS) is a major secretion pathway for virulence factors [23] and was first discovered in 1996. PcrV is a key needle-tip protein of the T3SS and plays a critical role in enabling the translocator proteins PopB/PopD to form pores in the host cell membrane, allowing the translocation of effector proteins into host cells [24]. As an essential component of the T3SS, PcrV represents an important target for virulence-blocking strategies. Studies have shown that PcrV is crucial for a functional T3SS and host cell intoxication; deletion of the *pcrV* gene (ΔPcrV) results in mutants that fail to deliver T3SS toxins into eukaryotic cells, thereby eliminating cytotoxicity both in vitro and in vivo [25,26]. Furthermore, an mRNA vaccine encoding the PcrV antigen has been shown to induce stronger antigen-specific immune responses, reducing bacterial loads and alleviating inflammation in burn and systemic infection models [27]. In another study on a hybrid protein containing PcrV and ExoS, formulated with alum and MPL adjuvant, immunized mice displayed robust humoral, mucosal, and cellular immune responses; significantly enhanced opsonophagocytic activity of antibodies; and reduced bacterial loads in the bladder and kidneys [28]. Collectively, these findings indicate that PcrV represents a promising antigen for a *Pseudomonas aeruginosa* vaccine, as compared with many other candidate antigens.

As PcrV is a protective antigen of *Pseudomonas aeruginosa*, its efficient delivery via the outer membrane vesicle (OMV) platform remains constrained by the low protein expression level of existing expression vectors in host bacteria, making it difficult to meet the requirements for recombinant OMV preparation. Therefore, there is an urgent need to construct novel high-expression vectors to achieve PcrV presentation in OMVs. Moreover, OMVs possess dual functions as both antigen carriers and natural adjuvants. The recombinant OMV strategy, in which PcrV is genetically engineered to be presented on OMVs, may differ from the strategy of using OMVs as an adjuvant mixed with free PcrV in terms of the associated immune responses, immune bias, and protective efficacy. The recombinant OMV approach enables co-delivery of the antigen and adjuvant, which theoretically induces stronger specific immune responses. In contrast, the mixing strategy, owing to the differential in vivo distribution of the antigen and adjuvant, may fail to maximize synergistic effects. Comparing these two strategies could provide critical insights for the rational design of OMV-based vaccines against *P. aeruginosa*.

Based on the above background, in this study, we first redesigned and constructed an expression vector for high-level expression of the PcrV protein, then successfully prepared engineered outer membrane vesicles presenting the PcrV antigen (recombinant OMV_PcrV_). Furthermore, we systematically compared the protective efficacy of two immunization strategies—recombinant OMV_PcrV_ versus the OMV adjuvant combined with PcrV protein—in inducing humoral immunity, cellular immunity, and resistance to *Pseudomonas aeruginosa* infection, with the aim of laying an experimental foundation for the development of novel *P. aeruginosa* vaccines.

## 2. Materials and Methods

### 2.1. Reagents, Experimental Animals, and Bacterial Strains

Luria–Bertani (LB) medium was purchased from OXOID Ltd. (Basingstoke, UK). Ethylenediaminetetraacetic acid (EDTA) solution (0.5 M), ELISA stop solution, one-component TMB chromogen solution, gentamycin sulfate, Anti-His Tag (HRP) monoclonal antibody, and 1× PBS buffer were obtained from Beijing Solarbio Science & Technology Co., Ltd. (Beijing, China). SWE rapid high-resolution electrophoresis buffer, ice-bath-free rapid transfer buffer, and protein-free rapid blocking solution were purchased from Wuhan Servicebio Technology Co., Ltd. (Wuhan, China). PVDF membranes were obtained from Immobilon^®^ (Merck Millipore Ltd., Burlington, MA, USA). BSA (Fatty Acid & IgG Free, BioPremium, Shanghai, China) and the BCA Protein Assay Kit (Enhanced) were purchased from Beyotime Biotechnology (Shanghai, China). The E.Z.N.A.^®^ Gel Extraction Kit and E.Z.N.A.^®^ Plasmid DNA Mini Kit I were purchased from Omega Bio-Tek, Inc. (Norcross, GA, USA). The PAGE Gel Fast Preparation Kit was obtained from Shanghai Epizyme Biomedical Technology Co., Ltd. (Shanghai, China). BL21(DE3) competent cells were purchased from Sangon Biotech Co., Ltd. (Shanghai, China). Electroporation cuvettes (2 mm gap) were purchased from BTX Harvard Apparatus (Holliston, MA, USA). The *Pseudomonas aeruginosa* PAO1 strain was kindly provided by Nankai University (Tianjin, China). The pBBRMCS5 plasmid was maintained in our laboratory. Six- to eight-week-old male and female specific-pathogen-free (SPF) mice were purchased from Beijing Gudou Jintai Biotechnology Co., Ltd. (Beijing, China).

### 2.2. Major Instruments

The major instruments used in this study are as follows: biosafety cabinet (Thermo Fisher Scientific, Waltham, MA, USA); ultracentrifuge (Hitachi, Ltd., Tokyo, Japan); flow cytometer (Thermo Fisher Scientific, Waltham, MA, USA); PCR thermal cycler (Dongsheng Biotech, Beijing, China); tissue homogenizer (Wuhan Servicebio Technology Co., Ltd., Wuhan, China); vertical protein electrophoresis system (Bio-Rad Laboratories, Hercules, CA, USA); chemiluminescence imager (Wuhan Servicebio Technology Co., Ltd., Wuhan, China); peristaltic pump (Avantor, Inc., Radnor, PA, USA); 0.22 μm filter bottle (Corning Incorporated, Corning, NY, USA).

### 2.3. Construction and Identification of the Recombinant Plasmid

Based on the pBBRMCS5 plasmid vector, primers flanking the *pcrV* and OprFI genes were designed and synthesized. The target gene fragments were amplified via PCR, double-digested with EcoRI and XbaI, and then ligated into the linearized vector using T4 DNA ligase to generate the recombinant plasmid. This plasmid was first transformed into BL21(DE3) competent cells. After verification by means of PCR and Western blotting, the plasmid was introduced into *Pseudomonas aeruginosa* PAO1 via electroporation. Finally, a genetically engineered *P. aeruginosa* strain stably expressing the target protein was obtained, as confirmed by means of PCR and Western blotting.

### 2.4. Extraction and Characterization of Recombinant Outer Membrane Vesicles from Pseudomonas aeruginosa

Stocks of PAO1(pBBRMCS5-*pRpL-pcrV*), PAO1(pBBRMCS5-*OprFI-pcrV*), and PAO1 (glycerol stock) were streaked onto LB agar plates containing gentamicin and incubated statically at 37 °C for strain activation. A single colony was picked and inoculated into 5 mL of LB liquid medium, then cultured with shaking at 180 rpm and 37 °C for 12–14 h to obtain the primary seed culture. Subsequently, 1% of the primary culture was transferred into 30 mL of LB liquid medium and cultured under the same conditions for 8–10 h to obtain the secondary seed culture. Again, 1% of the secondary seed culture was transferred into 1 L of LB liquid medium and cultured at 37 °C with shaking at 180 rpm for 14–16 h. After cultivation, ethylenediaminetetraacetic acid (EDTA) was added to the bacterial culture to a final concentration of 10 mM, followed by incubation on ice for 2–3 h. EDTA chelates divalent cations that maintain outer membrane stability, thereby promoting vesicle release. The bacterial suspension was centrifuged at 7000× *g* for 30 min at 4 °C, and the supernatant was collected. The supernatant was filtered through a 300 kDa aqueous membrane and concentrated to approximately 100 mL. The concentrated supernatant was aliquoted into ultracentrifuge tubes and centrifuged at 120,000× *g* for 2 h at 4 °C. The supernatant was discarded, and the pellet was resuspended in 500 μL of 1× PBS, filtered through a 0.22 μm filter for sterilization, collected in sterile 1.5 mL microcentrifuge tubes, and stored at 4 °C. The morphology and particle size of the obtained recombinant OMVs and OMVs were characterized by means of transmission electron microscopy (TEM) and nanoparticle tracking analysis (NTA). The expression of the recombinant protein in the vesicles was detected by means of Western blotting.

### 2.5. Animal Immunization with Recombinant Outer Membrane Vesicles from Pseudomonas aeruginosa

Six- to eight-week-old specific-pathogen-free (SPF) Balb/c mice were randomly divided into five groups (*n* = 15 per group): a PcrV protein group, an OMV + PcrV group, an OMV_PcrV_ group, an OMV group, and a PBS control group. The OMVs used in the OMV + PcrV group were wild-type outer membrane vesicles not loaded with the PcrV antigen (derived from PAO1 harboring the empty vector). Based on a pre-experimental evaluation of safety at different doses (data shown in Section 3.3), a dose of 20 μg was selected for formal immunization. The immunization doses were set as follows: mice in the protein immunization group received 5 μg of protein per mouse, and mice in the vesicle immunization groups received 20 μg of vesicles per mouse. To enhance the immune response, a booster immunization was administered on day 21 after the primary immunization, for a total of two immunizations. During the immunization period, the mice were regularly monitored for body weight changes and clinical signs, which were then recorded.

### 2.6. P. aeruginosa Challenge and Evaluation of Immune Protection

To ensure an accurate challenge dose, the actual colony-forming units (CFU) of the PAO1 strain were first determined via the serial dilution method using LB agar plates. Fresh bacterial culture was centrifuged at 10,000× *g* for 3 min, and the bacterial pellet was collected and resuspended in 1 mL of normal saline (0.9% NaCl). On day 42 post-immunization, the mice were challenged via two routes—subcutaneous injection (1 × 10^8^ CFU, corresponding to 10 times the median lethal dose) and intranasal instillation (1 × 10^8^ CFU)—to evaluate the immune protective efficacy.

### 2.7. Detection of Serum Antibody Levels in Balb/c Mice

Peripheral blood was collected from the mice on days 14 and 28 post-immunization. After being left to stand at room temperature for 2 h, the blood was centrifuged at 4000× *g* for 20 min at 4 °C, and the supernatant was collected. The supernatant was then centrifuged again at 8000× *g* for 5 min at 4 °C, and the resulting supernatant was collected as serum samples.

Specific antibody levels in the serum were measured by means of enzyme-linked immunosorbent assay (ELISA). Briefly, 96-well plates were coated with PcrV protein at a concentration of 5 μg/mL (100 μL/well) and incubated overnight at 4 °C. After three washes with PBST, the plates were blocked with 1% BSA solution for 2 h at 37 °C, followed by one further wash with PBST. Serum samples were diluted 1:100 and added to the antigen-coated wells (100 μL/well), then incubated for 1 h at 37 °C. The wells were washed three times with PBST. Subsequently, HRP-conjugated goat anti-mouse IgG, IgG1, IgG2a, and IgM secondary antibodies (diluted 1:5000) were added (200 μL/well) and incubated for 1 h at 37 °C, followed by five washes with PBST. Then, 100 μL of TMB chromogen solution was added to each well, and the plates were incubated at room temperature in the dark. After color development, 100 μL of stop solution was added to each well to terminate the reaction. The absorbance at 450 nm (OD value) was measured using a microplate reader.

### 2.8. Detection of Bacterial Load in Mouse Tissues

Twelve hours after the challenge, the mice were anesthetized and euthanized by means of cervical dislocation. Blood and lung, spleen, and liver tissues were collected under aseptic conditions and weighed. Each tissue was placed in 1 mL of sterile normal saline (0.9% NaCl) and homogenized using a tissue homogenizer. Serial dilutions of the homogenates and blood samples were prepared, plated onto LB agar plates, and incubated at 37 °C for 16–18 h. Colonies were then counted, and the number of viable bacteria in each tissue and blood sample was calculated.

### 2.9. Flow Cytometry Analysis

Mouse lung tissues were collected and rinsed with PBS to remove residual blood. The tissues were minced and placed in EP tubes containing 1 mg/mL digestive enzyme (prepared in DMEM and filtered through a 0.22 μm filter). The tubes were incubated in a shaking water bath at 37 °C with agitation at 250–300 rpm for 1 h. After digestion was largely complete, an equal volume of complete medium (DMEM supplemented with 10% FBS) was added to terminate the reaction. The cell suspension was filtered through a 70 μm cell strainer. The filtrate was centrifuged at 600× *g* for 10 min to collect the cell pellet. Three to five volumes of red blood cell lysis buffer were added; the cells were gently pipetted to mix them and lysed for 1–2 min. Subsequently, the suspension was centrifuged at 500× *g* for 5 min, and the supernatant was discarded. The pellet was resuspended in PBS and washed twice, followed by another centrifugation and removal of the supernatant. The cells were resuspended in 100 μL of PBS, and 1 μL each of antibody staining solution PE-CD4 (clone GK1.5, BioLegend, San Diego, CA, USA) and APC-CD8a (clone 53-6.7, BioLegend, San Diego, CA, USA) was added. After mixing, the cells were incubated in the dark for 30 min. Following centrifugation and removal of the supernatant, the cells were washed once with PBS and finally resuspended in 500 μL of PBS for flow cytometry analysis. Lymphocytes were gated by FSC-A/SSC-A, and 10,000 events were collected using a CytoFLEX cytometer (Beckman Coulter, Brea, CA, USA). CD3^+^ cells were gated prior to CD4^+^/CD8^+^ analysis. Fluorescence compensation was applied with single-stained controls. Isotype-matched antibodies (rat IgG2b κ for CD4; rat IgG2a κ for CD8; BioLegend) served as negative controls. To assess cytokine production in lung T cells, immunized mice were challenged with 1 × 10^6^ CFU of PAO1 and euthanized on day 3 post-infection [29]. After lung cells were harvested as described above, the cell suspension was layered onto Percoll solution and centrifuged, followed by red blood cell lysis. Following surface staining, cells were washed with Perm/Wash buffer and incubated in the dark with a cocktail of intracellular antibodies against TNF-α (clone MP6-XT22, BioLegend, San Diego, CA, USA), IFN-γ (clone XMG1.2, BioLegend, San Diego, CA, USA) and IL-17A (clone TC11-18H10.1, BioLegend, San Diego, CA, USA). Finally, cells were resuspended in FACS staining buffer for flow cytometric analysis.

## 3. Results and Analysis

### 3.1. Identification of the Recombinant Plasmid

**Figure 1:**
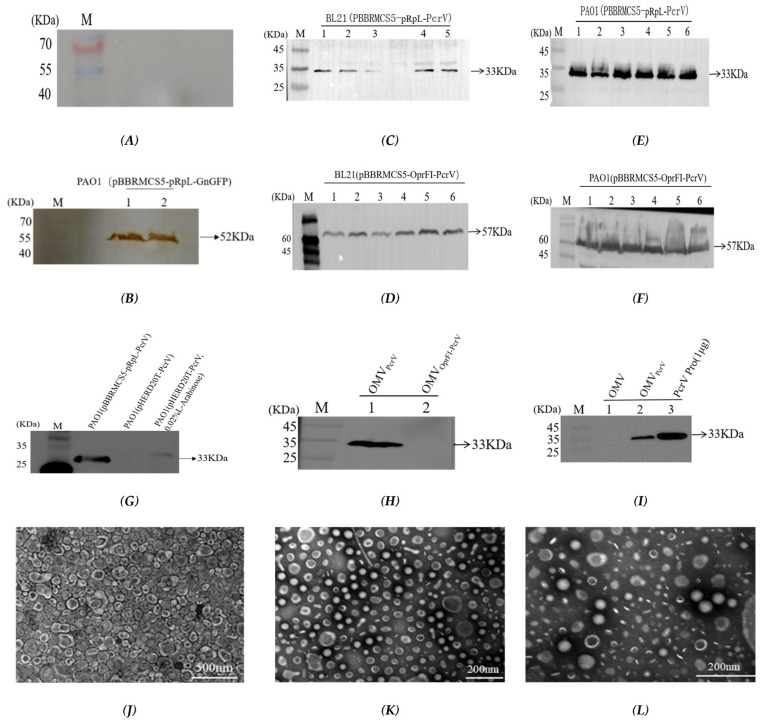
Extraction and characterization of outer membrane vesicles and recombinant outer membrane vesicles. (**A**) Protein expression of pBBRMCS5 (Lac) vector in PAO1 strain. (**B**) Protein expression of pBBRMCS5 (*pRpL*) vector in PAO1 strain. (**C**) Protein expression of recombinant plasmid pBBRMCS5-*pRpL-pcrV* in *Escherichia coli* BL21. (**D**) Protein expression of recombinant plasmid pBBRMCS5-*OprFI-pcrV* in *Escherichia coli* BL21. (**E**) Protein expression of recombinant plasmid pBBRMCS5-*pRpL-pcrV* in PAO1 strain. (**F**) Protein expression of recombinant plasmid pBBRMCS5-*OprFI-pcrV* in PAO1 strain. (**G**) Comparison of protein expression levels between pBBRMCS5 and pHERD20T vectors in PAO1 strain. (**H**) Total Western blot identification of recombinant outer membrane vesicles OMV_OprFI-PcrV_ and OMV_PcrV_. (**I**) Grayscale quantitative analysis of OMV_PcrV_ band of OMV_PcrV_ in recombinant outer membrane vesicles. Lane 1: wild-type outer membrane vesicles (OMV); Lane 2: recombinant outer membrane vesicles OMV_PcrV_; Lane 3: PcrV protein (1 μg). (**J**) Transmission electron microscopy image of wild-type OMV (×25.0 k magnification). (**K**) Transmission electron microscopy image of recombinant (×50.0 k magnification). (**L**) Transmission electron microscopy image of recombinant OMV_OprFI-PcrV_ (×80.0 k magnification).

Given the generally low expression levels observed with *Pseudomonas aeruginosa* expression vectors, the pBBRMCS5 vector was selected as a novel expression system in this study. A recombinant plasmid, pBBRMCS5-*GnGFP*, was constructed using green fluorescent protein (GFP) as a reporter gene and transformed into *P. aeruginosa*. The results showed that the GnGFP fusion protein was either undetectable or expressed at very low levels (Figure 1A). Therefore, the original lac promoter in the vector was replaced with tandem *pR* and *pL* promoters. Using this modified expression system in *P. aeruginosa*, a specific band of approximately 52 kDa, consistent with the expected size, was successfully detected (Figure 1B). Based on the modified vector, recombinant plasmids pBBRMCS5- *pRpL*-*pcrV* and pBBRMCS5-*OprFI-pcrV* were constructed and transformed into Escherichia coli BL21(DE3) competent cells. PCR identification (Figure S1) and Western blot analysis revealed specific bands of 33 kDa (PcrV) and 57 kDa (OprFI-PcrV) (Figure S2) (Figure 1C,D). These recombinant plasmids were then introduced into *P. aeruginosa* PAO1 by means of electroporation (Figure S3). Using the same identification methods, specific bands of the corresponding molecular weights were also detected (Figure 1E,F), confirming successful transfer of the recombinant plasmids into the PAO1 strain and effective expression of the target proteins (Figure S4). A comparison of the modified pBBRMCS5 vector with the commonly used *P. aeruginosa* vector pHERD20T showed that the modified vector had significantly increased protein expression. Grayscale analysis using ImageJ (version 1.54g) software indicated that the target protein expression intensity was approximately 24.5-fold higher than that in the control group (Figure 1G).

### 3.2. Characterization of Recombinant Outer Membrane Vesicles Delivering PcrV Antigen (OMV_PcrV_)

Recombinant and non-recombinant OMVs were extracted by means of ultracentrifugation. Western blot analysis revealed a specific band for PcrV at approximately 33 kDa, whereas the OprFI-PcrV fusion protein was not detected (negative result, Figure 1H), confirming that PcrV was successfully expressed in the outer membrane vesicles. A 40 μg sample of recombinant OMVs was loaded, and 1 μg of purified PcrV protein was used as a reference. Grayscale analysis using ImageJ software showed that the PcrV protein signal accounted for 22.4% of the reference purified PcrV signal (Figure 1I).

Transmission electron microscopy observation revealed that wild-type OMVs, recombinant OMV_PcrV_, and OMV_OprFI-PcrV_ (Figure 1J–L) all exhibited typical spherical vesicular structures with clear boundaries (Figure S5).

Nanoparticle tracking analysis using a Zeta View (Particle Metrix GmbH, Inning am Ammersee, Germany) instrument showed that the OMVs had a median particle size (D50) of 130.1 ± 4.2 nm and a particle concentration of (6.56 ± 0.24) × 10^10^ particles/mL; recombinant OMV_PcrV_ had a median particle size of 127.4 ± 5.3 nm and a particle concentration of (4.5 ± 1.5) × 10^10^ particles/mL (Table 1).

### 3.3. Safety Evaluation of Recombinant Outer Membrane Vesicles and Virulence Changes for the Engineered Strain

**Figure 2:**
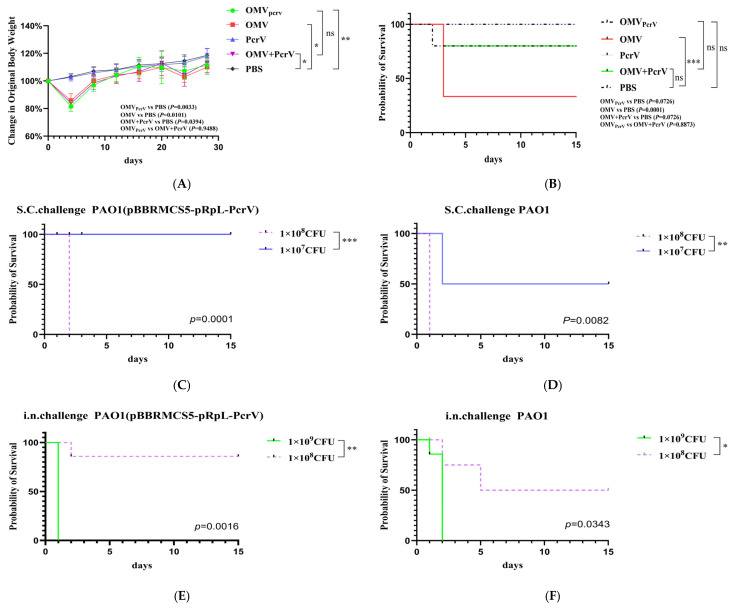
The safety of OMV_PcrV_ and a comparison of virulence between wild-type and engineered *P. aeruginosa* strains. Mice were immunized with PBS, empty OMVs, OMV + PcrV, or OMV_PcrV_ (all at a dose of 40 μg). (**A**) Body weight changes during the immunization period. (**B**) The survival of each group during the immunization period. To investigate the effect of plasmid transformation on bacterial virulence, an infection model was established. Mice were challenged with the engineered strain PAO1(pBBRMCS5-*pRpL-pcrV*) via the subcutaneous route (**C**) or the intranasal route (**E**), or with wild-type PAO1 via the subcutaneous route (**D**) or the intranasal route (**F**). Statistical significance was determined via the log-rank (Mantel–Cox) test (*n* = 4). ns, not significant; * *p* < 0.05; ** *p* < 0.01; *** *p* < 0.001.

To evaluate the safety of the recombinant outer membrane vesicles from *Pseudomonas aeruginosa*, a preliminary experiment was first conducted using a dose of 40 μg. The results showed that in the early stage of immunization, mice in the OMV and OMV_PcrV_ groups exhibited a significant decrease in body weight compared with the PBS control group, with gradual recovery starting on day 4 post-immunization (Figure 2A). Immunization with outer membrane vesicles led to varying degrees of mortality in the mice (Figure 2B): the mortality rate was 66.7% in the OMV group, whereas it decreased to 20% in both the OMV_PcrV_ group and the OMV + PcrV group. Evaluation using a bacterial infection model showed that the lethal dose of the engineered strain in mice infected via the subcutaneous route (Figure 2C) or the intranasal route (Figure 2E) was lower than that of the wild-type strain infected via the corresponding routes (Figure 2D,F), suggesting reduced toxicity of the engineered bacterial suspension.

**Figure 3:**
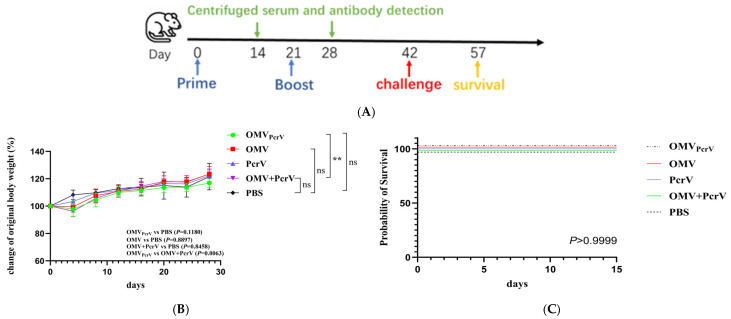
Immunization schedule, body weight changes, and survival during the immunization period. (**A**) A schematic of the immunization and challenge schedule. Mice were primed on day 0 and boosted on day 21. Serum was collected on days 14 and 28. On day 42, mice were challenged with *P. aeruginosa*, and survival was monitored for 14 days. (**B**) Body weight changes during the immunization period, expressed as a percentage of initial body weight (mean ± SD, *n* = 15 per group). Statistical significance for differences between groups was determined via two-way ANOVA with Tukey’s post hoc test. (**C**) Mouse survival during the immunization period (prior to challenge). No mortality occurred in any group, and survival probabilities did not differ significantly (log-rank test, *p* > 0.9999). ns, not significant; ** *p* < 0.01.

Preliminary experiments showed that a 40 μg dose of outer membrane vesicles caused mortality in mice. For safety reasons, the immunization dose was reduced to 20 μg in this study to evaluate its in vivo biosafety. Body weight monitoring (Figure 3A) revealed that mice in the OMV group experienced transient body weight loss in the early post-immunization period but began to recover from day 4 onwards, consistent with the dynamic changes observed in the 40 μg dose group. During the observation period, no mortality occurred in the 20 μg dose groups (Figure 3B), and the mortality rate in the vesicle-immunized groups decreased from 20% at the 40 μg dose to 0%, indicating that a moderate reduction in the immunization dose significantly improves the in vivo biosafety of recombinant outer membrane vesicles in mice.

### 3.4. Immune Protection Against P. aeruginosa Challenge and Detection of Bacterial Load in Mouse Tissues

**Figure 4:**
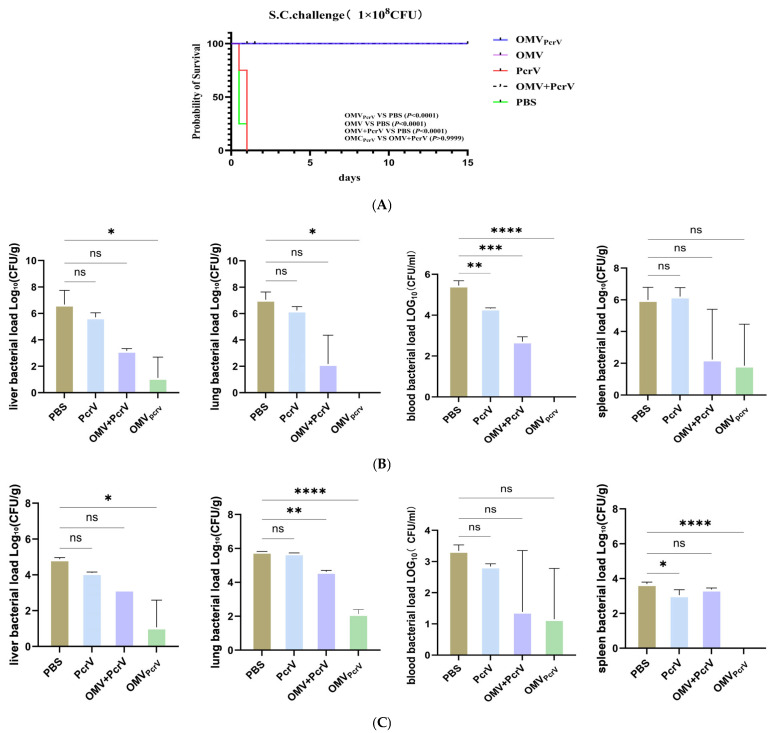
Protective efficacy of vaccination and analysis of bacterial burden in tissues. (**A**) Survival curves of mice after subcutaneous challenge. Bacterial loads in tissues (lung, liver, spleen) and blood at 12 h post-challenge: (**B**) subcutaneous infection; (**C**) intranasal infection. Data are expressed as colony-forming units (CFU) per gram of tissue or milliliter of blood in LOG_10_ format and presented as mean ± standard deviation (mean ± SD). ns: not significant (*p* ≥ 0.05); * *p* < 0.05; ** *p* < 0.01; *** *p* < 0.001; **** *p* < 0.0001. Mean ± SD (*n* = 3 mice per group); one-way ANOVA with Tukey’s test.

On day 42 post-immunization, mice in each group were challenged with *P. aeruginosa* PAO1 at a dose of 1 × 10^8^ CFU via either subcutaneous injection or intranasal instillation. In the subcutaneous infection model, the OMV, OMV_PcrV_, and OMV + PcrV groups all displayed 100% immune protection (all mice survived), whereas mice in the PBS control group and the PcrV protein group all died within 24 h after the challenge (Figure 4A). Bacterial load analysis showed that no viable bacteria (below the detection limit) were detected in the blood or lungs of mice in the OMV_PcrV_ immunized group, and the bacterial loads in the liver and spleen were lower than those observed for the PBS control group (Figure 4B). In the intranasal challenge model, the OMV immunized group exhibited reduced bacterial loads in all tested tissues (blood, spleen, liver, and lungs) compared with the PBS control group (Figure 4C). Some intergroup differences did not reach statistical significance, which may be attributed to the relatively high dispersion of individual data points within the experimental groups and biological heterogeneity in individual mouse responses.

### 3.5. Antibody Responses Induced by the Recombinant Outer Membrane Vesicle OMV_PcrV_

**Figure 5:**
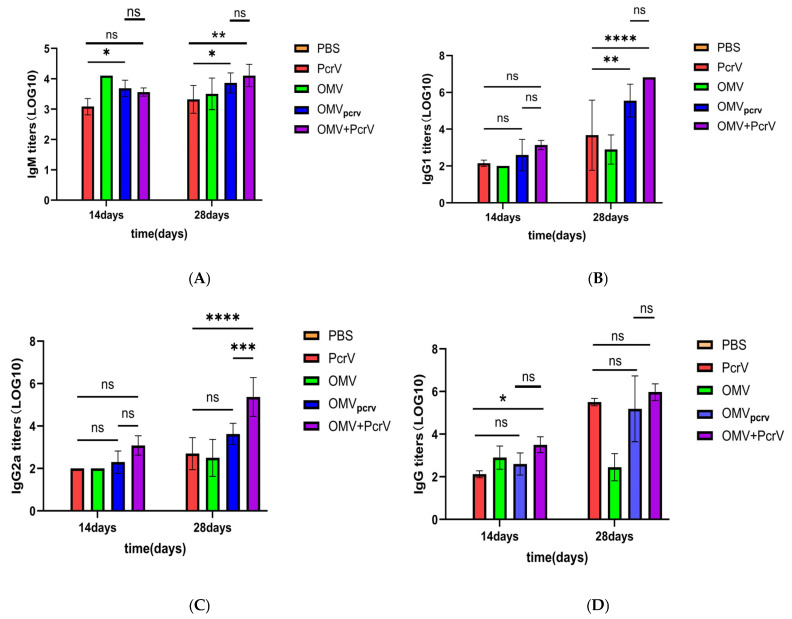
Specific antibody responses in mouse serum after immunization. Note: Serum was collected on day 14 and day 28 after intramuscular immunization. Levels of specific antibodies in serum were detected via enzyme-linked immunosorbent assay (ELISA), including (**A**) IgM, (**B**) IgG1, (**C**) IgG2a, and (**D**) IgG. ns: not significant (*p* ≥ 0.05); *: *p* < 0.05; **: *p* < 0.01; ***: *p* < 0.001; ****: *p* < 0.0001. Mean ± SD (*n* = 5 mice per group); two-way ANOVA with Tukey’s test.

To evaluate the enhancing effect of OMVs on the immunogenicity of the PcrV antigen, serum levels of IgM, IgG1, IgG2a, and total IgG antibodies were measured by means of ELISA using PcrV antigen-coated plates on days 14 and 28 post-immunization. The results showed that on day 14 post-immunization, the titers of IgM and total IgG in the OMV group were significantly higher than those in the PcrV group (Figure 5A), whereas no statistically significant differences in IgG1 or IgG2a levels were observed among the groups. On day 28 post-immunization, the IgG1 titers in both the OMV_PcrV_ and OMV + PcrV groups were significantly higher than that in the PcrV group, with no significant difference in IgG1 levels between the former two groups (Figure 5B). Additionally, both the OMV_PcrV_ and OMV + PcrV groups showed elevated IgG2a titers, with the highest IgG2a level observed in the OMV + PcrV group (Figure 5C). Furthermore, the total IgG levels in the OMV_PcrV_ and OMV + PcrV groups were comparable to those in the PcrV protein group (Figure 5D).

### 3.6. Histopathological Examination

**Figure 6:**
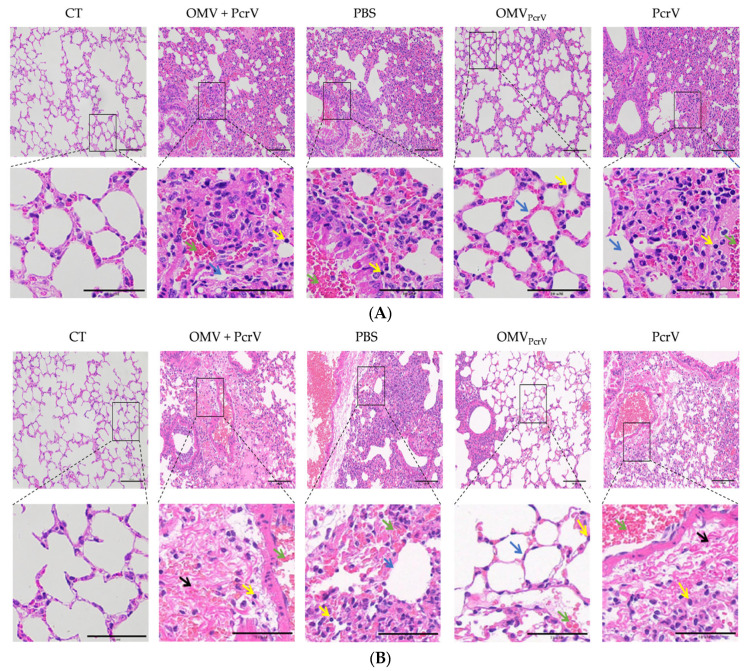
Histopathological changes in mouse lung tissues after subcutaneous and intranasal challenge. Note: HE staining; (**A**): subcutaneous challenge; (**B**): intranasal challenge. Scale bars: 100 μm (upper whole-field images), 50 μm (lower magnified images). Blue arrows, thickened alveolar septa; yellow arrows, inflammatory cell infiltration; green arrows, erythrocyte exudation; black arrows, disrupted alveolar structure, Dashed rectangles and lines denote the selected areas for magnified lower images.

Following the PAO1 challenge, mouse lung tissues were collected and subjected to H&E staining to evaluate pathological changes. In the subcutaneous infection model (Figure 6A), the PBS and PcrV groups exhibited typical acute pneumonic changes: diffuse thickening of the alveolar septa (blue arrows), extensive inflammatory cell infiltration (yellow arrows) and erythrocyte extravasation (green arrows), and collapse of the alveolar structure. In contrast, the OMV_PcrV_ group showed only a few scattered inflammatory cells with intact alveolar architecture, indicating the best protective effect. In the intranasal infection model (Figure 6B), the PBS group displayed the most severe lesions, characterized by diffuse congestion and edema, inflammatory infiltration, and disruption of the alveolar structure (black arrows). The PcrV group still showed severe inflammation and hemorrhage. The OMV + PcrV group exhibited reduced lesions, although interstitial thickening and exudation remained. The OMV_PcrV_ group showed a regular alveolar morphology with only minimal inflammatory cells, demonstrating superior protective efficacy compared with the other groups.

### 3.7. OMV_PcrV_ Induces T-Cell Responses in Mouse Lungs

**Figure 7:**
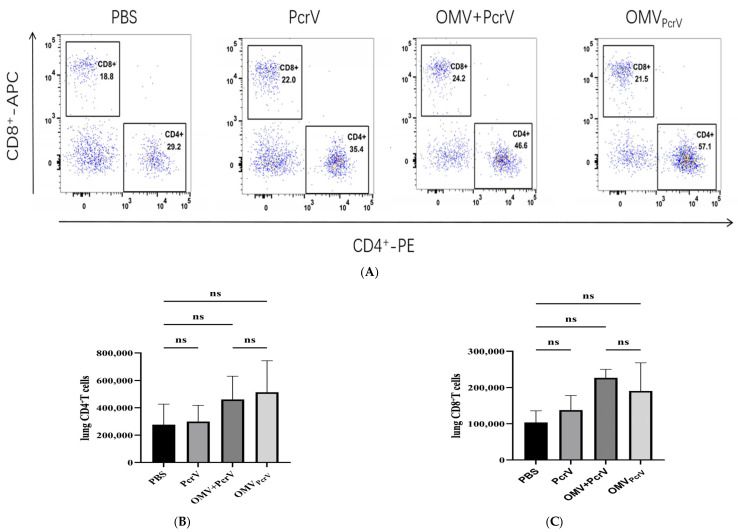

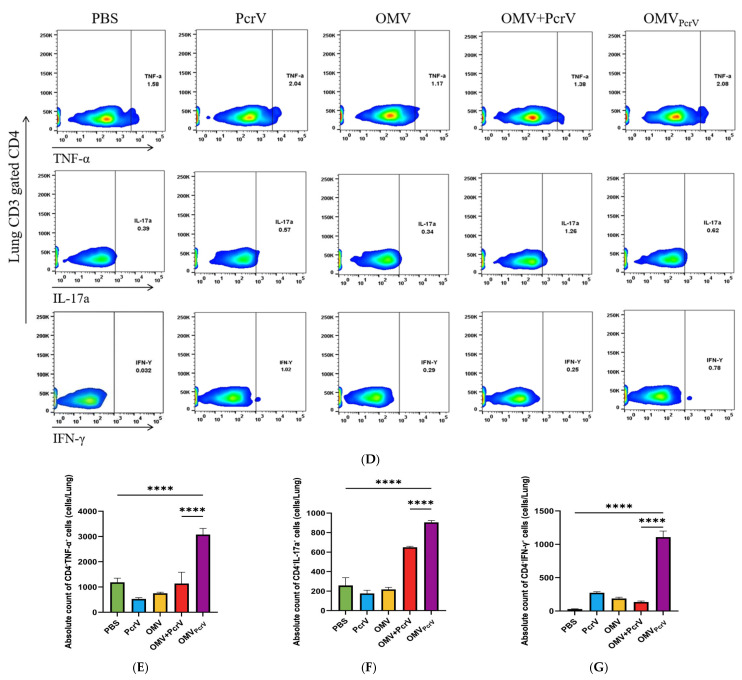
Analysis of CD4^+^ and CD8^+^ T-cell subsets and cytokine production in lung tissues of immunized mice. (**A**) Representative flow cytometry plots showing distribution of CD4^+^ and CD8^+^ T cells in lung tissues from different immunization groups. (**B**) Quantitative statistics of CD4^+^ T cells in lung tissues of each group. (**C**) Quantitative statistics of CD8^+^ T cells in lung tissues of each group. (**D**) Representative flow cytometry plots of cytokine-producing cells in lung tissues following in vivo stimulation with PAO1 strain. (**E**–**G**) Quantitative analysis of cytokine-positive cells in lung tissues of each group. ns, no significant difference (*p* ≥ 0.05); ****: *p* < 0.0001. Data are presented as mean ± SD, with three mice per group. Statistical analysis was performed using one-way ANOVA followed by Tukey’s multiple comparisons test. bar colors: green = PBS group, blue = PcrV group, yellow = OMV group, red = OMV + PcrV group, purple = OMV_PcrV_ group.

To evaluate the effects of different immunization strategies on T-cell subsets in mouse lung tissues, we first measured the percentages and absolute counts of CD4^+^ and CD8^+^ T cells by means of flow cytometry. The results showed that, compared with the PBS control group, all immunization groups (PcrV, empty OMV, OMV + PcrV, and OMV_PcrV_) exhibited increased percentages and absolute counts of both CD4^+^ and CD8^+^ T cells in the lungs (Figure 7A); however, no statistically significant differences were observed among the groups (*p* ≥ 0.05). Among these, the OMV_PcrV_ group showed a trend toward higher CD4^+^ T-cell counts (Figure 7B), and the OMV + PcrV group showed slightly increased CD8^+^ T-cell counts (Figure 7C), but neither reached statistical significance. Based on the above findings, we further characterized the secretion profiles of TNF-α, IL-17A, and IFN-γ. Flow cytometric analysis (Figure 7D) revealed that the abundances of TNF-α (Figure 7E), IL-17A (Figure 7F), and IFN-γ (Figure 7G) cells in lung tissues were markedly elevated in the OMV_PcrV_ group compared with the PBS control, sole PcrV, empty OMV, and OMV + PcrV mixture groups (*p* < 0.0001). Collectively, no obvious discrepancies were observed in the total counts of pulmonary CD4^+^ and CD8^+^ T cells across all groups. Nevertheless, the OMV_PcrV_ vaccine potently elicited robust Th1 and Th17 cellular immune responses.

## 4. Discussion

*P. aeruginosa* is a leading cause of healthcare-associated infections, and the growing challenge of multidrug resistance has outpaced antibiotic development [30,31,32]. Vaccines targeting PcrV, a component of the type III secretion system, have shown promise but are often limited by poor antigen stability and weak cellular immune responses [33,34,35]. Vaccine development is also constrained by antigenic diversity, biofilm-mediated immune evasion, host immunological abnormalities, and poorly defined protective mechanisms [36,37]. Outer membrane vesicles (OMVs) offer a versatile platform: they act as natural immunostimulants and can be engineered to display heterologous antigens, enabling co-delivery of antigen and adjuvant [38,39]. In this study, we engineered a recombinant OMV delivering PcrV (OMV_PcrV_) and compared it with empty OMVs and a formulation of free PcrV protein (OMV + PcrV). We found that OMV_PcrV_ significantly enhanced bacterial clearance and tissue protection, whereas both OMV_PcrV_ and OMV + PcrV provided 100% survival protection following lethal PAO1 challenge.

Th17 cells and their effector cytokine IL-17A play a central role in mucosal immunity against respiratory infections, mediating the clearance of multiple extracellular bacterial pathogens by recruiting neutrophils, inducing antimicrobial peptide production, and strengthening the epithelial barrier [40,41,42,43]. The superiority of OMV_PcrV_ over the OMV + PcrV mixture was most evident in reducing bacterial load. In the OMV_PcrV_ group, no viable bacteria were detected in the blood or lung tissue, and bacterial loads in the liver and spleen were also lower than those in the mixed group. Histopathology further confirmed that the OMV_PcrV_ group preserved alveolar architecture with only mild inflammation, whereas the mixed group showed moderate tissue damage. These protective outcomes correlated closely with the induction of Th17-type mucosal immunity. Sereme et al. [44] demonstrated in an auxotrophic live attenuated *Pseudomonas aeruginosa* vaccine model that antigen-specific Th17/IL-17A responses induced by airway mucosal im45munization, together with mucosal IgA, are critical for protection against lung infection, and that this protection is strictly dependent on the mucosal route. Kuipers et al. [45] used *Salmonella* OMVs to display high densities of a heterologous pneumococcal antigen on the surface; intranasal immunization successfully elicited antigen-specific IL-17A responses and significantly reduced nasal colonization without the need for additional adjuvants. Together, these findings support the notion that recombinant antigen display on OMVs can effectively elicit protective Th17-biased mucosal immunity, thereby enhancing immune protection against *P. aeruginosa* infection.

Both OMV_PcrV_ and OMV + PcrV induced a mixed Th1/Th2 antibody response (elevated IgG1 and IgG2a) and generated comparable numbers of CD4^+^ and CD8^+^ T cells in the lung tissue. Notably, although total T-cell counts did not differ significantly among the groups, OMV_PcrV_ induced significantly higher levels of TNF-α, IFN-γ, and IL-17A than were observed for all other groups. Among these, the Th17 response is particularly important for pulmonary defense against *P. aeruginosa*. This view has recently been reinforced by independent studies showing that the magnitude of the pulmonary Th17 response following immunization with a live attenuated whole-cell mucosal vaccine or a plant virus-based PcrV vaccine [46] directly correlates with protection against lethal PAO1 challenge in mice. The OMV + PcrV mixed strategy also elevated these cytokine-positive T-cell populations, but to a lesser extent. Thus, OMV_PcrV_ not only preserves but may even enhance functional cellular immune responses.

The safety assessment revealed a dose-dependent profile. At a dose of 40 μg, the mortality rate in the OMV-alone group was 66.7%, whereas that in the OMV_PcrV_ group decreased to 20%. At the 20 μg dose used throughout this study, no deaths occurred in any group. This toxicity is largely attributable to lipopolysaccharide (LPS) and other endogenous OMV components. Previous studies have shown that genetic detoxification of LPS—for example, by deleting the lipid A acyltransferase gene *lpxL1* or *msbB*—can markedly reduce the intrinsic toxicity of OMVs without fully compromising their adjuvant activity [47,48]. Our ongoing work is exploring such modifications to improve the therapeutic window of OMV_PcrV_.

We acknowledge that the challenge experiments in this study used only the PAO1 reference strain. However, protective efficacy against clinical isolates was examined in our previous work [49]. Furthermore, a recent study on a STING-OMV nanovaccine reported that an OMV-based platform conferred complete protection against the hypervirulent PA14 clinical isolate, and passive serum transfer experiments demonstrated cross-protection against the heterologous PAO1 strain [50], suggesting that the OMV platform may inherently possess some protective potential. Second, we did not measure mucosal IgA or opsonophagocytic activity, nor did we evaluate the durability of protection beyond 42 days. In addition, all experimental data were derived from mouse models, and intrinsic differences in immune system composition and response characteristics between mice and humans warrant cautious interpretation in the translation of our findings to humans. These parameters and model limitations are important for a full characterization of immune protection. Nevertheless, the marked difference in bacterial clearance between OMV_PcrV_ and OMV + PcrV, together with the Th17-biased immune response, provides strong experimental evidence that antigen-delivering OMVs represent a superior strategy.

## 5. Conclusions

In summary, this study revealed differences between the OMV_PcrV_ and OMV + PcrV strategies in terms of bacterial clearance and the induction of Th17 immune responses. OMV_PcrV_ showed superior performance in these respects, although the underlying immune mechanisms remain to be elucidated. Nevertheless, these observations provide an experimental basis for OMV-based antigen delivery strategies.

## Figures and Tables

**Table 1:** Characterization of concentration and particle size of OMVs.

	OMVs	OMV_PcrV_
Diluted Concentration	(1.64 ± 0.06) × 10^8^ particles/mL	(1.5 ± 0.5) × 10^8^ particles/mL
Dilution Factor	400	300
Original Concentration	(6.56 ± 0.24) × 10^10^ particles/mL	(4.5 ± 1.5) × 10^10^ particles/mL
Median (D50)	130.1 ± 4.2 nm	127.4 ± 5.3 nm

Note: Concentration values represent post-dilution measured particle concentrations. Original concentrations were back-calculated from detected values and corresponding dilution factors. Median values indicate the particle size median, with D50 denoting the median particle size. Note: Data are presented as the mean ± standard deviation (SD) of three independent experiments.

## Data Availability

The original contributions presented in this study are included in the article/Supplementary Material. Further inquiries can be directed to the corresponding authors.

## Supplementary Materials

The following supporting information can be downloaded at: https://www.mdpi.com/article/10.3390/vaccines14060518/s1, Figure S1: PCR amplification of target gene fragments from recombinant plasmids pBBRMCS5-*pRpL-pcrV*, pBBRMCS5-OprFI-PcrV, and pHERD20T-PcrV. Figure S2: Western blot analysis of PcrV and OprFI-PcrV fusion protein expression in recombinant *E. coli* BL21(DE3). Figure S3: PCR amplification of target gene fragments from recombinant plasmids pBBRMCS-PcrV, pBBRMCS5-OprFI-PcrV, and pHERD20T-PcrV. Figure S4: Western blot analysis of PcrV and OprFI-PcrV fusion protein expression in recombinant *Pseudomonas aeruginosa* PAO1. Figure S5: Transmission electron microscopy images of OMVs and recombinant OMV_PcrV_/OMV_OprFI-PcrV_ vesicles.

## Abbreviations

The following abbreviations are used in this manuscript:

OMV	Outer membrane vesicle
PcrV	Type III secretion system needle-tip protein PcrV
T3SS	Type III secretion system
LPS	Lipopolysaccharide
PAMPs	Pathogen-associated molecular patterns
PRRs	Pattern recognition receptors
APCs	Antigen-presenting cells
CFU	Colony-forming units
NTA	Nanoparticle tracking analysis
TEM	Transmission electron microscopy
ELISA	Enzyme-linked immunosorbent assay
PCR	Polymerase Chain Reaction
EDTA	Ethylenediaminetetraacetic acid
PBS	Phosphate-Buffered Saline
HRP	Horseradish Peroxidase
TMB	Tetramethylbenzidine
FBS	Fetal Bovine Serum
SPF	Specific pathogen-free
s.c.	Subcutaneous
i.n.	Intranasal
i.m.	Intramuscular
