# Supplementary material for: A Recombinant OMV-Based Vaccine Elicits Potent Protective Immunity Against Pseudomonas aeruginosa
Source: Vaccines (Basel). 2026 Jun 9;14(6):518. doi: 10.3390/vaccines14060518 (PMC13307874; doi:10.3390/vaccines14060518)
Supplement: Supplementary file 1 [file vaccines-14-00518-s001.zip › vaccines-4309424-supplementary.pdf]

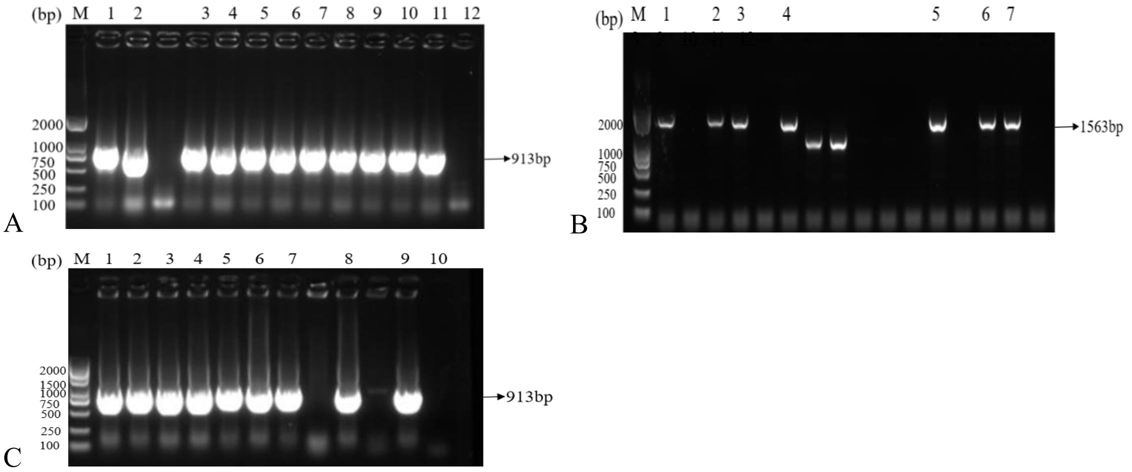

Figure S1.Amplification of OprFI-PcrV and PcrV gene fragments.Note: **Panel A**: Amplification of the PcrV fragment from recombinant plasmid pBBRMCS5-pRpL-PcrV; Lane M: DL2000 DNA Marker; Lanes 1–8**:** Amplified PcrV fragments; Lane 9: Negative control. **Panel B**: Amplification of the target fragment from recombinant plasmid pBBRMCS5-pRpL-OprFI-PcrV; Lane M: DL2000 DNA Marker; Lanes 1–4: Amplified OprFI-PcrV fragments. **Panel C**: Amplification of the PcrV fragment from recombinant plasmid pHERD20T-PcrV; Lane M: DL2000 DNA Marker; Lanes 1–9: Amplified PcrV fragments; Lane 10: Negative control.

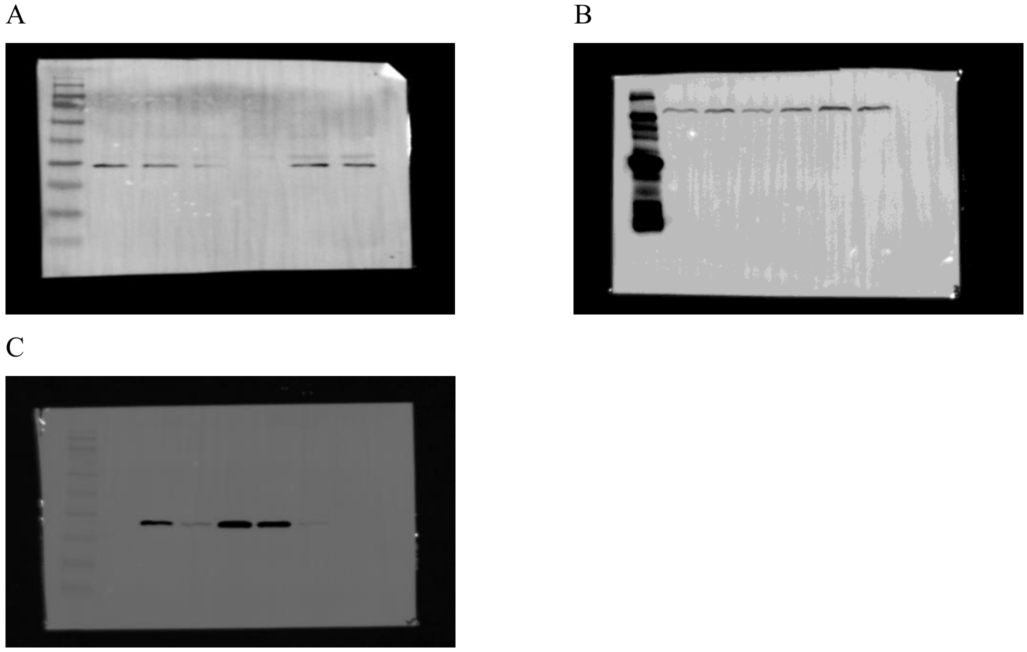

Figure S2.Detection of the target protein expression in Escherichia coli by Western blot.Note: **Panel A** shows the PcrV protein. Lane M: Protein relative molecular weight marker (10-180 KDa); Lanes 1-5: Bacterial suspension of BL21(pBBRMCS5-pRpL-PcrV). **Panel B** shows the OprFI-PcrV protein. Lane M: Protein relative molecular weight marker (10-180 KDa); Lanes 1-5: Bacterial suspension of BL21(pBBRMCS5-pRpL-OprFI-PcrV). **Panel C** shows the PcrV protein. Lane M: Protein relative molecular weight marker (10-180 KDa); Lanes 1-5: Bacterial suspension of BL21(pHERD20T-PcrV).

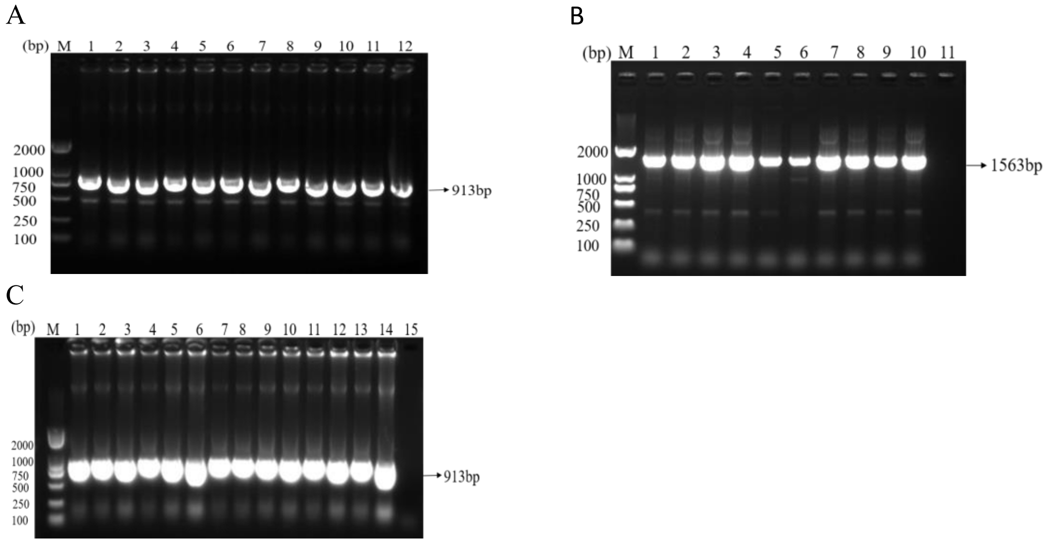

Figure S3.Amplification of OprFI-PcrV and PcrV gene fragments.Note: **Panel A** shows the amplification of the PcrV fragment from the pBBRMCS5-pRpL-PcrV plasmid. Lane M: DL2000 DNA Marker; Lanes 1–12: Amplified PcrV fragments. **Panel B** shows the amplification of the OprFI-PcrV fragment from the pBBRMCS5-pRpL-OprFI-PcrV plasmid. Lane M: DL2000 DNA Marker; Lanes 1–10: Amplified OprFI-PcrV fragments; Lane 11: Negative control. **Panel C** shows the amplification of the PcrV fragment from the pHERD20T-PcrV plasmid. Lane M: DL2000 DNA Marker; Lanes 1–11: Amplified PcrV fragments; Lane 12: Negative control.

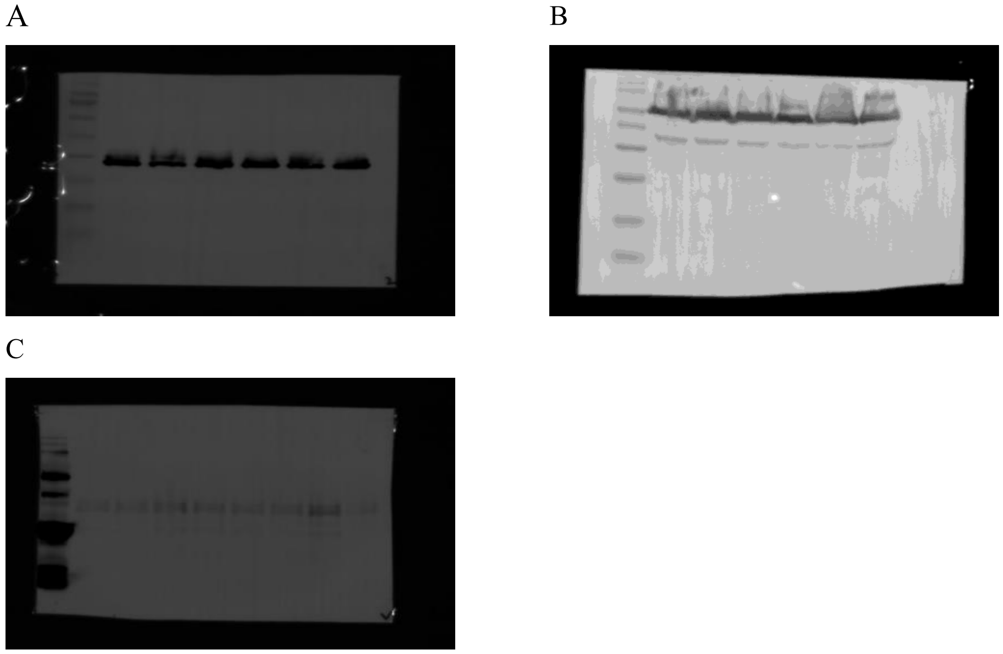

Figure S4.Western blot analysis of target protein expression in *Pseudomonas aeruginosa*.Note: **Panel A**: PcrV protein. Lane M: Protein molecular weight marker (10–180 kDa); Lanes 1–6: *P. aeruginosa* PAO1 (pBBRMCS5-PRPL-PcrV) bacterial culture.**Panel B**: OprFI-PcrV protein. Lane M: Protein molecular weight marker (10–180 kDa); Lanes 1–6: *P. aeruginosa* PAO1 (pBBRMCS5-PRPL-OprFI-PcrV) bacterial culture.**Panel C**: PcrV protein. Lane M: Protein molecular weight marker (10–180 kDa); Lanes 1–6: *P. aeruginosa* PAO1 (pHERD20T-PcrV) bacterial culture.

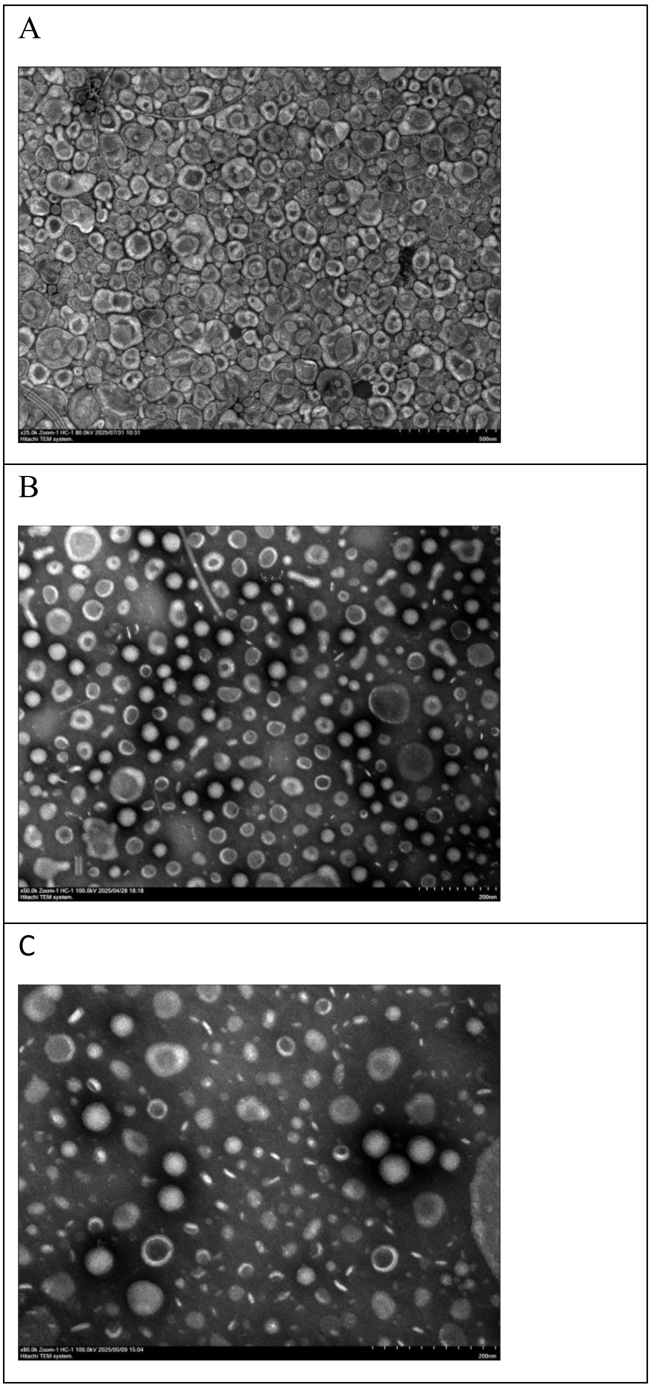

Figure S5.TEM images of OMV and recombinant OMV.Note: **Panel A**: Image of OMV sample (×25.0k); **Panel B**: Image of OMV_{PcrV} sample (×50.0k); **Panel C**: Image of $OMV_{OprFI\text{-}PcrV}$ sample (×80.0k).
